# Does Interhospital Transfer Influence the Outcomes of Patients Receiving Surgery for Acute Type A Aortic Dissection? Type A Aortic Dissection: Is Transfer Hazardous or Beneficial?

**DOI:** 10.1155/2019/5692083

**Published:** 2019-03-18

**Authors:** Yuan-Hsi Tseng, Chih-Chen Kao, Chien-Chao Lin, Chien-Wei Chen, Ming-Shian Lu, Chu-Hsueh Lu, Yao-Kuang Huang

**Affiliations:** ^1^Division of Thoracic and Cardiovascular Surgery, Chiayi Chang Gung Memorial Hospital, Chiayi and Chang Gung University College of Medicine, Taoyuan, Taiwan; ^2^Department of Diagnostic Radiology, Chang Gung Memorial Hospital, Chang Gung University, Chiayi and Taoyuan, Taiwan

## Abstract

**Introduction:**

The progression of acute type A aortic dissection may cause immediate death, such that, in the event of its diagnosis, emergency surgery is indicated. Relatedly, an interhospital transfer may prolong the time from diagnosis to surgery. This study therefore investigated how interhospital transfers impact surgical outcomes for acute type A aortic dissection.

**Materials and Methods:**

After excluding those patients who received deferred surgery for acute type A aortic dissection, 112 patients who received emergency surgery for the condition at our hospital from January 2011 to January 2018 were enrolled. These patients were divided into two groups, one consisting of the patients who were sent directly to our emergency department (group 1) and the other consisting of the patients who were transferred from another hospital after first being diagnosed with type A aortic dissection (group 2). The collected data included the patient demographics, clinical characteristics, operative findings and methods, postoperative outcomes, latest follow-up time, and most recent status.

**Results:**

There were 59 patients in group 1 and 53 patients in group 2. Univariate analysis revealed that group 1 had significantly more patients with a previous stroke (*p* = 0.007). Moreover, the average length of time from receiving a computed tomography (CT) scan to entering the operating room (OR) was shorter for the group 1 patients (*p* < 0.001). However, except for the incidence of postoperative acute kidney injury (14.5% versus 33.3%,* p* = 0.024), there was no statistical difference between the groups in terms of the operative findings and outcomes, such as hypotension before cardiopulmonary bypass, hemopericardium, other complications, and survival rate. Multivariate analysis showed that the independent predictors of hospital mortality included age > 61.5 years (*p* = 0.017), respiratory rate upon admission > 18.5 breaths/minute (*p* = 0.046), and total bypass time > 265.6 minutes (*p* = 0.015). For the patients who survived to discharge, log-rank analysis demonstrated similar cumulative survival rates for the two groups (*p* = 0.62). Further multivariate analysis showed that the risk of death after discharge was associated with the interval between the CT scan and OR entry (hazard ratio = 0.97 per minute; 95% confidence interval, 0.950–0.998;* p* = 0.037).

**Conclusion:**

In this study, it was found that interhospital transfer did not influence the surgical outcomes of patients with acute type A aortic dissection. As such, it can be concluded that the transfer of the patients with type A aortic dissection to tertiary hospitals with experienced cardiac surgical teams may not increase the surgical risk.

## 1. Introduction

Acute type A aortic dissection is a lethal disease because it may progress to hemopericardium, cardiac tamponade, or severe aortic regurgitation. The immediate mortality rate is 1% per hour, and the mortality rate is 50% within the first 24 hours [1, 2]. Moreover, the condition may also cause various neurological and vascular sequalae. Therefore, emergency surgery is considered the gold standard for treatment.

However, while cardiac surgeons are available at all hours in tertiary hospitals, it is possible for a diagnosis of acute type A aortic dissection to be established at a nontertiary hospital. As such, patients with the condition may need to be transferred in some cases. At the same time, an interhospital transfer is time-consuming, and considering the dynamics of dissection, the deterioration of a patient's condition during transfer is expected [3].

Our hospital is a tertiary referral hospital; thus, we often receive patients with acute type A aortic dissection who have been transferred from nearby hospitals. In this study, we analyzed the outcomes of these patients and compared them to those for patients who were both diagnosed and treated at our hospital.

## 2. Materials and Methods

### 2.1. Patients

The protocol for this retrospective study was approved by the Institutional Review Board of Chang Gung Memorial Hospital (CGMF IRB No. 201801700B0). Because the patient data were retrospective and were kept anonymous, the need for signed informed consent was waived for this study.

The data for 120 consecutive patients who underwent ascending aortic replacement at our hospital from January 2011 to January 2018 were initially collected. Patients were then excluded if they received surgery for an ascending aortic aneurysm, the surgery for acute type A aortic dissection was deferred to another day, or the type A aortic dissection was subacute or chronic. Finally, 112 patients were enrolled in this study.

This study assessed the intraoperative findings and outcomes of 2 separate patient groups. If a patient first visited our hospital for both examination and treatment, that patient was included in group 1. If a patient was first diagnosed with acute type A aortic dissection at another hospital and then transferred to our hospital for treatment, that patient was included in group 2. There were 59 patients in group 1 and 53 patients in group 2.

The collected data for each patient included his or her age, sex, comorbidities, admission vital signs, preoperative status, interval between receiving a computed tomography (CT) scan and entering the operating room (OR) (hereafter referred to as the interval between CT and OR), operative findings and methods, survival, and complications. Hypotension was defined as systolic blood pressure < 80 mmHg.

Furthermore, in order to survey those patients who died during transport, we reviewed the 2010-to-2017 data regarding out-of-hospital cardiac arrests retrieved from our emergency department database.

### 2.2. Interhospital Transfer Decision and Communication

In the telephone communications regarding a potential interhospital transfer, there are several questions that we routinely ask:

1. What are the main diagnosis and associated medical history of the patient?
2. What are the patient's vital signs and current clinical condition?
3. Why can the original hospital's doctors not treat the patient?
4. Does the patient's family know his or her diagnosis and condition?
5. Does the family understand the risks entailed by an interhospital transfer?

Our emergency team will then ask our surgical team, including a perfusionist, surgical intensive care unit staff, and operating room team members, whether it is possible, based on the answers to the above questions, to treat the patient immediately or not. If a decision to transfer the patient is made, the patient is transported to our hospital with a medical summary and any associated images. Because our hospital does not have a helipad, all patients transferred to our hospital use land transport.

### 2.3. Statistical Analysis

In the comparison of the baseline and clinical characteristics, the aortic vessel involvement, operative findings and procedures, and postoperative outcomes of group 1 and group 2, continuous variables are presented as medians and interquartile ranges and categorical variables are shown as numbers and percentages. The Mann-Whitney* U* test and chi-square test (or Fisher's exact test) were used to compare the two groups via univariate analysis.

However, considering that the baseline characteristics may have caused bias in estimating the risks of hemopericardium, hypotension before cardiopulmonary bypass, mortality, and surgical complications for the two groups, the odds ratios were adjusted for those baseline characteristics that were significantly different between the two groups in the univariate analysis using the Mantel-Haenszel method. In applying the Mantel-Haenszel method, the continuous variables which reached statistically significant differences were transformed into dichotomous variables; the cut-off points of these continuous variables were determined by receiver operating characteristic (ROC) curves. Moreover, when analyzing the impact of postoperative complications, the patients who died within 24 hours after surgery were excluded because such deaths were presumably highly related to surgical factors.

Among nine patients who died within 24 hours after surgery, five died in the operating theater: three of them died because of failure to wean off cardiopulmonary bypass and the rest died because of rupture of graft-aorta anastomoses during rewarming. Another four patients died in the intensive care unit of surgery: two died because of severe myocardial failure, one suffered from acute respiratory failure, and one suffered from refractory bleeding after the Bentall procedure.

In analyzing the risk of hospital mortality, only variables that had* p* < 0.05 in the univariate analysis were considered in the multivariate analysis. Similarly, only the continuous variables which reached statistical differences were transformed into dichotomous variables for multivariate analysis with ROC curves. The multivariate analysis was calculated with logistic regression.

Finally, for the patients who survived to discharge, a survival analysis was conducted using the Kaplan-Meier method and log-ranked test, while a multivariate analysis was calculated with the Cox proportional hazard model.

Statistical analyses were conducted using SPSS for Windows (Version 17.0; SPSS, Inc., Chicago, IL, USA) and Microsoft Excel 2003.

## 3. Results

From 2010 to 2017, there were 3 patients with acute type A aortic dissection who died during transport, one of whom was involved in a severe traffic accident. The mortality rate during transport was 5.36%.

As shown in Table 1, the univariate analysis results showed that the group 1 patients had a higher rate of previous stroke (16.9% versus 1.9%,* p* = 0.07) and a shorter interval between CT and OR (median, 87 versus 141 minutes,* p* < 0.001). There were no significant differences between the two groups in terms of age, sex, other comorbidities, admission vital signs, or the rate of intubation in the emergency department.

In the CT images, aortic arch and abdominal vessel involvement (dissection, severe stenosis, or occlusion) was not uncommon. 25 patients had unilateral carotid artery (or innominate artery) involvement, and four of them showed carotid artery occlusion or severe stenosis. 22 patients had bilateral carotid artery (or innominate artery plus left common carotid artery) involvement, and one of them had right common carotid artery occlusion. In respect of abdominal vessel involvement, 13 patients suffered from celiac trunk involvement, and three of them showed total occlusion. 20 patients had superior mesenteric artery involvement, and two of them demonstrated occlusion. Besides, 14 patients had unilateral renal artery involvement, and seven of them suffered from renal artery occlusion or severe stenosis. Four patients had bilateral renal artery involvement, and two of them suffered from bilateral renal artery occlusion.

After excluding missing data, the comparison of the two groups' aortic vessel involvement showed no statistical difference (Table 2).

With respect to the operative findings (Table 3), the two groups did not have a statistically significant difference in terms of the proportion of patients with hypotension before cardiopulmonary bypass or hemopericardium. A comparison of the patient outcomes for the two groups (Table 4), meanwhile, indicated no significant differences between them in terms of survival rate or complications, with the exception of the rate of acute kidney injury (14.5% versus 33.3%,* p* = 0.024).

Because the baseline characteristics of previous stroke and interval between CT and OR showed significant differences between the two groups in the univariate analysis, the odds ratios of the operative findings (hypotension before cardiopulmonary bypass and hemopericardium) and outcomes (hospital mortality and major complications) were adjusted by previous stroke and interval between CT and OR > 155.5 minutes, respectively. Although group 2 seemed to have higher risks of hemopericardium (adjusted odds ratios, 1.51 and 1.61) and hospital mortality (adjusted odds ratios, 1.93 and 2.21), these differences were not significant (Table 5).

According to the univariate analysis, the preoperative and operative predictors of hospital mortality included older age (*p* = 0.002), a history of coronary artery disease (*p* = 0.045), not smoking (*p* = 0.021), a higher respiratory rate upon admission (*p* = 0.008), intubation at the emergency department (*p* = 0.02), hypotension before cardiopulmonary bypass (*p* = 0.037), hemopericardium (*p* = 0.008), intimal tear at the ascending aorta or arch (*p* = 0.021), the extent of replacement limited to the ascending aorta ± hemiarch (*p* = 0.047), higher total bypass time (*p* = <0.001), higher aortic cross-clamping time (*p* = 0.009), and the use of deep hypothermic circulatory arrest for brain protection during open distal anastomosis (*p* = 0.002). According to the multivariate analysis, the independent predictors were age > 61.5 years (*p* = 0.017), respiratory rate upon admission > 18.5 breaths/minute (*p* = 0.046), and total bypass time > 265.6 minutes (*p* = 0.015) (Table 6).

**Figure 1:**
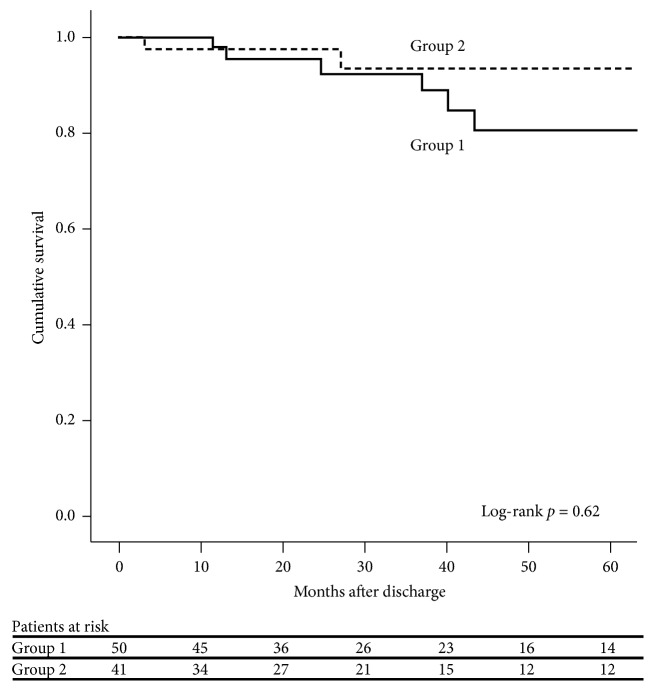
The Kaplan-Meier survival curve of the patients who survived to hospital discharge.

For the 91 patients who survived until hospital discharge, the log-rank test indicated that the two groups did not have a significant difference between their survival curves (log-rank test,* p* = 0.62; Figure 1). A multivariate analysis including interhospital transfer, history of previous stroke, the interval between CT and OR, and postoperative acute kidney injury in the model was then conducted, and only the interval between CT and OR was associated with death after discharge (hazard ratio = 0.97 per minute; 95% confidence interval, 0.950–0.998;* p* = 0.037).

## 4. Discussion

The diameter and extent of aortic dissection usually progress with time [4]. Relatedly, the increase in mortality has been found to be significant within the first two weeks after onset, especially when organ ischemia occurs [5, 6]. As such, we originally presumed that patients transferred from other hospitals would have higher rates of hemopericardium and hypotension and worse outcomes due to the progression of aortic dissection. Nonetheless, the results of this study showed that there was no difference between such patients and those diagnosed and treated at a single hospital in terms of intraoperative findings or survival rate.

As expected, the patients transferred from other hospitals had a longer wait, on average, from CT scan to surgery than the patients who visited our emergency department initially. Several past articles discussing interhospital transfers for acute type A aortic dissection have implied that the time required to complete a transfer may be positively correlated to the surgical mortality [7–9]. However, in this study, we found no significant differences between the two patient groups in the duration of mechanical ventilator support, length of intensive care unit stay, or survival rate, with only the rate of postoperative acute kidney injury being significantly higher in group 2. Similarly, in the analysis of the follow-up outcomes, there was no difference in short- to middle-term survival between the two groups, even after adjustment for history of previous stroke, the interval between CT and OR, and postoperative acute kidney injury.

There are two factors that may have caused our results to differ from those of previous studies. First, in our study, the longest transfer duration by the ground-based emergency medical service (EMS) was only about 3 hours. The majority of the patients were transferred to our hospital within 90 minutes after telephone contact. The efficiency and quickness of these EMS transfers might thus be the main factor accounting for the roughly equal survival rates of the transferred patients and the nontransferred patients. Relatedly, the extremely short average delay from diagnosis to surgery might have played an important role in our results. Immediate surgery for type A aortic dissection has been defined as surgery within 24 hours of diagnosis [10]. Nonetheless, because surgery for acute type A aortic dissection is viewed as an absolutely emergent type of surgery in our hospital, our hospital requires that such a patient be sent to the operating theater within 30 minutes from the time of surgery registration. Therefore, after excluding the patients who received delayed surgery, we found that the majority of our immediate surgeries were performed within 4 hours after the establishment of the diagnosis. Therefore, our interval from image survey to surgery was superior to the standard for emergency surgeries in previous studies, which may be another reason for the roughly equal survival rates of the transferred patients and the nontransferred patients.

With respect to postoperative complications, the rate of acute kidney injury was higher in group 2. This may have been caused by a longer organ malperfusion time, on average, in group two 2 to the delays resulting from the transfers. Moreover, acute kidney injury is related to hospital mortality. Relatedly, early diagnosis with optimal treatment has been recommended to reduce the associated morbidity and mortality [11–13].

## 5. Study Limitations

This was a retrospective, single-institution study. In spite of the attendant limitations, we attempted to identify the impact of interhospital transfer on surgical risk. However, some factors that may influence patient outcomes were not included in this study, such as the time of onset of symptoms, the parameters of the primary transfer (that is, the transfer from the scene to the primary hospital), and preoperative medication use. Multi-institutional studies may provide further information allowing for the more accurate estimation of the risks of interhospital transfer.

## 6. Conclusion

Although patients with type A aortic dissection who must be transferred from one hospital to another may have their surgeries delayed, the present study found no differences between such patients and nontransferred patients in terms of intraoperative findings, including hypotension before cardiopulmonary bypass, hemopericardium, complications, and hospital mortality. As such, the study results indicate that the transfer of patients with type A aortic dissection to tertiary hospitals with experienced cardiac surgical teams may not pose an increased surgical risk.

## Figures and Tables

**Table 1:** Preoperative baseline and clinical characteristics.

	Group 1	Group 2	*p*
	(n = 59)	(n = 53)	
Age (in years)	67 (54–80)	65 (55.5–73)	0.27
Male/female	28/31	21/32	0.40
Comorbidities			
Diabetes mellitus, n (%)	8 (13.6)	12 (22.6)	0.23
Hypertension, n (%)	48 (81.4)	49 (92.5)	0.08
Hyperlipidemia, n (%)	8 (13.6)	3 (5.7)	0.16
Hepatitis B, n (%)	3 (5.1)	8 (15.1)	0.08
Hepatitis C, n (%)	1 (1.7)	5 (9.4)	0.10
Gout, n (%)	4 (6.8)	4 (7.5)	1.00
Coronary artery disease, n (%)	1 (1.7)	4 (7.5)	0.19
Chronic kidney disease, n (%)	10 (16.9)	3 (5.7)	0.06
Prior stroke, n (%)	10 (16.9)	1 (1.9)	*0.007*
COPD, n (%)	2 (3.4)	2 (3.8)	1.00
Smoking, n (%)	17 (28.8)	17 (32.1)	0.71
Admission vital signs			
SBP (mmHg)	121 (91–148)	138 (109.5–157)	0.11
DBP (mmHg)	68 (57–80)	77 (63–91.5)	0.12
HR (beats/min)	73 (58–89)	74 (64–87.5)	0.34
RR (/min)	19 (18–20)	20 (18–20)	0.62
Hypotension at ED, n (%)	11 (18.6)	8 (15.1)	0.62
Intubation at ED, n (%)	3 (5.1)	7 (13.2)	0.19
Interval from CT to OR (min)	87 (72–115)	141 (119–195.25)	*<0.001*

COPD, chronic obstructive pulmonary disease; CT, computed tomography scan; DBP, diastolic blood pressure; ED, emergency department; HR, heart rate; OR, operating room; RR, respiratory rate; SBP, systolic blood pressure.

**Table 2:** Aortic vessel involvement.

	Group 1*∗*	Group 2*∗∗*	*p*
CCA or IA involvement			
One side	16 (27.1%)	9 (18.4%)	0.28
Two sides	12 (20.3%)	10 (20.4%)	0.99
CCA occlusion or severe stenosis	4 (6.8%)	1 (2.0%)	0.37
Celiac trunk involvement	8 (13.6%)	5 (9.8%)	0.54
Celiac trunk occlusion	0	3 (5.9%)	0.10
SMA involvement	10 (16.9%)	10 (19.6%)	0.72
SMA occlusion	0	2 (3.9%)	0.21
Renal artery involvement			
One side	6 (10.2%)	8 (15.7%)	0.39
Two sides	1 (1.7%)	3 (5.9%)	0.33
Renal artery occlusion or severe stenosis	3 (5.1%)	6 (11.8%)	0.30

CCA, common carotid artery; IA, innominate artery; SMA, superior mesenteric artery.

*∗*: patients who were sent directly to our emergency department.

*∗∗*: patients who were transferred from another hospital after first being diagnosed with type A aortic dissection.

**Table 3:** Operative findings and methods.

	Group 1	Group 2	*p*
	(n = 59)	(n = 53)	
Hypotension before CPB, n (%)	20 (33.9)	17 (32.1)	0.84
Hemopericardium, n (%)	25 (42.4)	26 (49.1)	0.48
Location of intimal tear			
Aortic root, n (%)	5 (8.5)	6 (11.3)	0.61
Ascending aorta, n (%)	25 (42.4)	21 (39.6)	0.77
Aortic arch, n (%)	8 (13.6)	8 (15.1)	0.82
AsAO to arch, n (%)	3 (5.1)	2 (3.8)	1.00
No visible tear, n (%)	18 (30.5)	16 (30.2)	0.97
Extent of replacement			
AsAO ± hemiarch, n (%)	51 (86.4)	49 (92.5)	0.30
Aortic root replacement, n (%)	4 (6.8)	3 (5.7)	1.00
Total arch, n (%)	4 (6.8)	1 (1.9)	0.37
Cardiopulmonary bypass			
TBT (min)	251 (213.5–317)	264 (230–299)	0.62
ACT (min)	132 (106–169.25)	128 (112–157.5)	0.79
CAT (min)	39 (31.75–50.5)	38 (33.5–46.5)	0.88
Brain protection			
ACP, n (%)	51 (86.4)	42 (79.2)	0.31
RCP, n (%)	6 (10.2)	9 (17)	0.29
DHCA, n (%)	2 (3.4)	2 (3.8)	1.00
Cardioplegia			0.30
Blood cardioplegia, n (%)	51 (86.4)	49 (92.5)	
HTK, n (%)	8 (13.6)	4 (7.5)	
Check bleeding, n (%)	8 (13.6)	4 (7.5)	0.30

ACP, antegrade cerebral perfusion; ACT, aortic cross-clamping time; AsAO, ascending aorta; CAT, cardiac arrest time; CPB, cardiopulmonary bypass; DHCA, deep hypothermic circulatory arrest; HTK, histidine-tryptophan-ketoglutarate solution; RCP, retrograde cerebral perfusion; SBP, systolic blood pressure; TBT, total bypass time.

**Table 4:** Postoperative outcomes.

	Group 1	Group 2	*p*
	(n = 59)	(n = 53)	
Survival > 24 hrs after surgery, n (%)	55 (93.2)	48 (90.6)	0.73
Temporary inotropic agent, n (%)	9 (16.4)	9 (18.8)	0.75
Complications			
New stroke, n (%)	12 (21.8)	8 (16.7)	0.51
Heart failure, n (%)	3 (5.5)	2 (4.2)	1.00
Respiratory failure, n (%)	15 (27.3)	14 (29.2)	0.83
Acute kidney injury, n (%)	8 (14.5)	16 (33.3)	*0.02*
Surgical wound infection, n (%)	4 (7.3)	2 (4.2)	0.68
Pneumonia, n (%)	22 (40)	21 (43.8)	0.70
GI bleeding, n (%)	3 (5.5)	6 (12.5)	0.30
Arrhythmia, n (%)	5 (9.1)	10 (20.8)	0.09
Survival to discharge, n (%)	50 (84.7)	41 (77.4)	0.32
Duration of MV support (day)	4 (2–9)	4 (2–10.5)	0.64
Length of ICU stay (day)	9 (5–17.75)	7 (5–18)	0.67
Length of hospital stay (day)	22 (15–40)	24 (14–39.5)	0.80

GI, gastrointestinal; ICU, intensive care unit; MV, mechanical ventilator.

**Table 5:** Adjusted odds ratios for the association between interhospital transfer and intraoperative findings and outcomes.

	Unadjusted odds ratio (95% CI)	Adjusted odds ratio^1^ (95% CI)	Adjusted odds ratio^2^ (95% CI)
Hypotension before CPB	0.92 (0.42–2.03)	0.94 (0.41–2.13)	1.29 (0.50–3.33)
Hemopericardium	1.31 (0.62–2.76)	1.51 (0.70–3.27)	1.61 (0.63–4.07)
Hospital mortality	1.63 (0.62–4.24)	1.93 (0.69–5.42)	2.21 (0.62–7.87)
Major complications*∗*	1.64 (0.78–3.47)	1.62 (0.73–3.61)	0.92 (0.34–2.46)

1: adjustment for prior stroke.

2: adjustment for interval between CT and OR > 155.5 minutes.

*∗*: major complications including new stroke, heart failure, respiratory failure, and acute kidney injury.

CI, confidence interval; CPB, cardiopulmonary bypass; CT, computed tomography scan; OR, operating room entrance.

**Table 6:** Multivariate analysis of preoperative and operative predictors for hospital death.

Variate	OR	*p*
Age > 61.5 years	21.96	0.02
RR > 18.5 breaths/min	6.45	0.046
TBT > 265.5 mins	13.42	0.01

Hosmer and Lemeshow test, *p* = 0.94.

OR, odds ratio; RR, respiratory rate; TBT, total bypass time.

## Data Availability

The data used to support the findings of this study are included within the article.
